# Supplementary material for: Multimodal decoding of human liver regeneration
Source: Nature. 2024 May 1;630(8015):158–65. doi: 10.1038/s41586-024-07376-2 (PMC11153152; doi:10.1038/s41586-024-07376-2)
Supplement: Supplementary file 1 — This file contains a list of Members and institutions participating in the Acute Liver Failure Study Group; legends to Supplementary Video files, Supplementary Tables and source data information [file 41586_2024_7376_MOESM1_ESM.docx]

**Multimodal decoding of human liver regeneration**

Matchett KP^1^, Wilson-Kanamori JR^1†^, Portman JR^1†^, Kapourani CA^1,2,3†^, Fercoq F^4^, May S^4^, Zajdel E^1^, Beltran M^1^, Sutherland EF^1^, Mackey JBG^4^, Brice M^1^, Wilson GC^1^, Wallace SJ^1^, Kitto L^1^, Younger NT^1^, Dobie R^1^, Mole DJ ^1,5^, Oniscu GC^6,7^, Wigmore SJ^1,5^, Ramachandran P^1^, Vallejos CA^2,8^, Carragher NO^9^, Saeidinejad MM^10^, Quaglia A^11,12^, Jalan R^10,13^, Simpson KJ^14^, Kendall TJ^1^, Rule JA^15^, Lee WM^15^, Hoare M^16,17^, Weston CJ^18,19^, Marioni JC^20,21,22^, Teichmann SA^22,23,24^, Bird TG^1,4^, Carlin LM^4,25^, Henderson NC^1,26^*

^1^Centre for Inflammation Research, Institute for Regeneration and Repair, University of Edinburgh, Edinburgh, UK.

^2^MRC Institute of Genetics and Cancer, University of Edinburgh, Edinburgh, UK.

^3^School of Informatics, University of Edinburgh, Edinburgh, UK.

^4^Cancer Research UK Beatson Institute, Glasgow, UK.

^5^University Department of Clinical Surgery, University of Edinburgh, Edinburgh, UK.

^6^Edinburgh Transplant Centre, Royal Infirmary of Edinburgh, Edinburgh, UK.

^7^Division of Transplant Surgery, CLINTEC, Karolinska Institutet, Stockholm, Sweden.

^8^The Alan Turing Institute, London, UK.

^9^Cancer Research UK Edinburgh Centre, Institute of Genetics and Cancer, University of Edinburgh, Edinburgh, UK.

^10^Institute for Liver and Digestive Health, University College London, London, UK.

^11^Department of Cellular Pathology, Royal Free London NHS Foundation Trust, London, UK.

^12^UCL Cancer Institute, University College London, London, UK.

^13^European Foundation for the Study of Chronic Liver Failure, Barcelona, Spain.

^14^Department of Hepatology, University of Edinburgh and Scottish Liver Transplant Unit, Royal Infirmary of Edinburgh, Edinburgh, UK.

^15^Department of Internal Medicine, University of Texas, Southwestern Medical Center, Dallas, TX, USA.

^16^Early Cancer Institute, University of Cambridge, Cambridge, UK.

^17^Department of Medicine, University of Cambridge, Cambridge, UK.

^18^NIHR Birmingham Biomedical Research Centre, University Hospitals Birmingham NHS Foundation Trust and University of Birmingham, Birmingham, UK.

^19^Institute of Immunology and Immunotherapy, University of Birmingham, Birmingham, UK.

^20^Cancer Research UK Cambridge Institute, University of Cambridge, Cambridge, UK.

^21^European Molecular Biology Laboratory, European Bioinformatics Institute, Cambridge, UK.

^22^Wellcome Sanger Institute, Wellcome Genome Campus, Hinxton, Cambridge, UK.

^23^European Molecular Biology Laboratory, European Bioinformatics Institute, Cambridge, UK.

^24^Department of Physics, Cavendish Laboratory, Cambridge, UK.

^25^School of Cancer Sciences, University of Glasgow, Glasgow, UK.

^26^MRC Human Genetics Unit, Institute of Genetics and Cancer, University of Edinburgh, Edinburgh, UK.

†These authors contributed equally.

*Address correspondence to:

Neil Henderson, Centre for Inflammation Research, Institute for Regeneration and Repair, University of Edinburgh, Edinburgh, UK. Phone: 0131.651.8150; Email: [Neil.Henderson@ed.ac.uk](mailto:Neil.Henderson@ed.ac.uk)

**Members and institutions participating in the Acute Liver Failure Study Group:**

W.M. Lee, M.D. (Principal Investigator), George A. Ostapowicz, M.D., Frank V. Schiødt, M.D., Julie Polson, M.D., University of Texas Southwestern, Dallas, TX; Anne M. Larson, M.D., University of Washington, Seattle, WA; Timothy Davern, M.D., University of California, San Francisco, CA; Michael Schilsky, M.D., Mount Sinai School of Medicine, NY, NY; Timothy McCashland, M.D., University of Nebraska, Omaha, NE; J. Eileen Hay, MBBS,

Mayo Clinic, Rochester, MN; Natalie Murray, M.D., Baylor University Medical Center, Dallas, TX; A. Obaid S. Shaikh, M.D., University of Pittsburgh, Pittsburgh, PA; Andres Blei, M.D. (deceased), Northwestern University, Chicago, IL; Atif Zaman, M.D., University of Oregon, Portland, OR; Steven H.B. Han, M.D., University of California, Los Angeles, CA; Robert Fontana, M.D., University of Michigan, Ann Arbor, MI; Brendan McGuire, M.D., University of Alabama, Birmingham, AL; Ray Chung, M.D., Massachusetts General Hospital,

Boston, MA; Alastair Smith, MB, ChB, Duke University Medical Center, Durham, NC; Robert Brown, M.D., Cornell/Columbia University, NY, NY; Jeffrey Crippin, M.D., Washington University, St. Louis, MO; Edwin Harrison, Mayo Clinic, Scottsdale, AZ; Adrian Reuben, MBBS, Medical University of South Carolina, Charleston, SC; Santiago Munoz, M.D., Albert Einstein Medical Center, Philadelphia, PA; Rajender Reddy, M.D., University of Pennsylvania, Philadelphia, PA; R. Todd Stravitz, M.D., Virginia Commonwealth University,

Richmond, VA; Lorenzo Rossaro, M.D., University of California Davis, Sacramento, CA; Raj Satyanarayana, M.D., Mayo Clinic, Jacksonville, FL; and Tarek Hassanein, M.D., University of California, San Diego, CA.

**Supplementary Video 1:**

Application of human migratory hepatocyte gene module to mouse hepatocyte nuclei across timepoints post APAP-induced acute liver injury.

**Supplementary Video 2:**

*Anxa2* gene expression in mouse hepatocyte nuclei across timepoints post APAP-induced acute liver injury.

**Supplementary Video 3:**

Application of SPATA-derived mouse central (left) and portal (right) zonation signatures to mouse hepatocyte nuclei across timepoints post APAP-induced mouse liver injury.

**Supplementary Video 4:**

Intravital imaging of mouse liver in *Hep;tdTom* reporter mice (hepatocytes express cytoplasmic tdTomato) demonstrating centrilobular hepatocyte necrosis in real-time from 24 hours post APAP-induced mouse liver injury. Scale bar 30µm.

**Supplementary Video 5:**

Intravital imaging of mouse liver in *Hep;tdTom* reporter mice (hepatocytes express cytoplasmic tdTomato) demonstrating centrilobular hepatocyte necrosis in real-time from 24 hours post APAP-induced mouse liver injury. Scale bar 30µm.

**Supplementary Video 6:**

Intravital imaging of mouse liver in *Hep;tdTom* reporter mice (hepatocytes express cytoplasmic tdTomato) demonstrating wound closure between 36-42 hours following APAP-induced mouse liver injury. Boxed area is magnified in SI Video 6b. Scale bar 30µm.

**Supplementary Video 7:**

Magnification of boxed area from SI Video 6a - intravital imaging of mouse liver in *Hep;tdTom* reporter mice (hepatocytes express cytoplasmic tdTomato) demonstrating wound closure between 36-42 hours following APAP-induced mouse liver injury. Scale bar 30µm.

**Supplementary Video 8:**

Intravital imaging of mouse liver in *Hep;tdTom* reporter mice (hepatocytes express cytoplasmic tdTomato) between 36-42 hours following APAP-induced mouse liver injury. White arrowheads denote hepatocytes with a motile morphology, including membrane ruffling and the formation of lamellipodia at the hepatocyte leading edge abutting the wound. Scale bar 5µm.

**Supplementary Video 9:**

Intravital imaging of mouse liver in *Hep;tdTom* reporter mice (hepatocytes express cytoplasmic tdTomato) between 36-42 hours following APAP-induced mouse liver injury. White arrowheads denote hepatocytes with a motile morphology, including membrane ruffling and the formation of lamellipodia at the hepatocyte leading edge abutting the wound. Scale bar 10µm.

**Supplementary Video 10:**

Intravital imaging of mouse liver in *Hep;tdTom* reporter mice (hepatocytes express cytoplasmic tdTomato) between 36-42 hours following APAP-induced mouse liver injury. White arrowheads denote hepatocytes with a motile morphology, including membrane ruffling and the formation of lamellipodia at the hepatocyte leading edge abutting the wound. Scale bar 5µm.

**Supplementary Video 11:**

Intravital imaging of mouse liver in *Hep;tdTom* reporter mice (hepatocytes express cytoplasmic tdTomato) between 36-42 hours following APAP-induced mouse liver injury. White arrowheads denote hepatocytes with a motile morphology, including membrane ruffling and the formation of lamellipodia at the hepatocyte leading edge abutting the wound. Scale bar 5µm.

**Supplementary Video 12:**

Intravital imaging of mouse liver in *Hep;tdTom* reporter mice (hepatocytes express cytoplasmic tdTomato) between 36-42 hours following APAP-induced mouse liver injury. White arrowheads denote hepatocytes with a motile morphology, including membrane ruffling and the formation of lamellipodia at the hepatocyte leading edge abutting the wound. Scale bar 5µm.

**Supplementary Video 13:**

Intravital imaging of mouse liver in *Hep;tdTom* reporter mice (hepatocytes express cytoplasmic tdTomato) between 36-42 hours following APAP-induced mouse liver injury. White arrowheads denote hepatocytes with a motile morphology, including membrane ruffling and the formation of lamellipodia at the hepatocyte leading edge abutting the wound. Scale bar 5µm.

**Supplementary Video 14:**

Intravital imaging of mouse liver in *Hep;tdTom* reporter mice (hepatocytes express cytoplasmic tdTomato) between 36-42 hours following APAP-induced mouse liver injury. White arrowheads denote hepatocytes with a motile morphology, including membrane ruffling and the formation of lamellipodia at the hepatocyte leading edge abutting the wound. Scale bar 5µm.

**Supplementary Table 1:**

Patient metadata (snRNAseq, ST, and multiplex smFISH).

**Supplementary Table 2:**

Gene signatures in human and mouse snRNA-seq and ST, and human multiplex smFISH.

**Supplementary Table 3:**

Marker genes for human and mouse snRNA-seq; p-values were computed using Wilcoxon Rank Sum tests.

**Supplementary Table 4:**

GO results for human and mouse snRNA-seq, and ST SPATA analysis; p-values were computed using hypergeometric tests.

**Supplementary Table 5:**

Interactome; p-values were computed using permutation tests.

**Supplementary Table 6:**

Antibodies and reagents.

**Source Data Fig. 2**

Source data for panels i, j.

**Source Data Fig. 3**

Source data for panels a, c, f, g.

**Source Data Fig. 4**

Source data for panels b, d, e.

**Source Data Fig. 7**

Source data for panels a, b, c, d, e, f, j, k.

**Source Data Fig. 8**

Source data for panels b, c, d, e, f, g, h, j.

**Source Data Fig. 9**

Source data for panels c, d, e, f, g, h, i, j, k, l.

**Source Data Fig. 10**

Source data for panels a, b, c, d.
