# Supplementary material for: Multimodal decoding of human liver regeneration
Source: Nature. 2024 May 1;630(8015):158–65. doi: 10.1038/s41586-024-07376-2 (PMC11153152; doi:10.1038/s41586-024-07376-2)
Supplement: Supplementary file 2 — Reporting Summary [file 41586_2024_7376_MOESM2_ESM.pdf]

nature portfolio

Corresponding author(s): Neil C. Henderson

Last updated by author(s): Mar 8, 2024

Reporting Summary

Nature Portfolio wishes to improve the reproducibility of the work that we publish. This form provides structure for consistency and transparency in reporting. For further information on Nature Portfolio policies, see our Editorial Policies and the Editorial Policy Checklist.

Statistics

For all statistical analyses, confirm that the following items are present in the figure legend, table legend, main text, or Methods section.

n/a	Confirmed	
☐	☒	The exact sample size (*n*) for each experimental group/condition, given as a discrete number and unit of measurement
☐	☒	A statement on whether measurements were taken from distinct samples or whether the same sample was measured repeatedly
☐	☒	The statistical test(s) used AND whether they are one- or two-sided<br>*Only common tests should be described solely by name; describe more complex techniques in the Methods section.*
☐	☒	A description of all covariates tested
☐	☒	A description of any assumptions or corrections, such as tests of normality and adjustment for multiple comparisons
☐	☒	A full description of the statistical parameters including central tendency (e.g. means) or other basic estimates (e.g. regression coefficient) AND variation (e.g. standard deviation) or associated estimates of uncertainty (e.g. confidence intervals)
☐	☒	For null hypothesis testing, the test statistic (e.g. *F*, *t*, *r*) with confidence intervals, effect sizes, degrees of freedom and *P* value noted<br>*Give P values as exact values whenever suitable.*
☒	☐	For Bayesian analysis, information on the choice of priors and Markov chain Monte Carlo settings
☒	☐	For hierarchical and complex designs, identification of the appropriate level for tests and full reporting of outcomes
☐	☒	Estimates of effect sizes (e.g. Cohen's *d*, Pearson's *r*), indicating how they were calculated

Our web collection on statistics for biologists contains articles on many of the points above.

Software and code

Policy information about availability of computer code

Data collection

Initial processing of single-nuclei RNA-sequencing data was performed using the commercial CellRanger pipeline (10X Genomics, version 3.1.0 (see methods)). Initial processing of spatial transcriptomics data was performed using the commerical SpaceRanger pipeline (10X Genomics, version 1.0.0 (see methods)). Multiplex smFISH data was processed via Resolve Biosciences. Subsequent analyses were performed using the open-source R programming language (version 3.4.1).
Fluorescent and brightfield microscopy images were acquired using Zen Blue software (Zeiss v2.6) on an Axioscan.Z1 instrument (Zeiss) or EVOS FL Auto 2. Flow cytometry data was acquired using a 5laser Fortessa cytometer (Becton Dickinson). Intravital microscopy images were acquired using LSM 880 NLO multiphoton microscope (Zeiss). Scratch wound data was acquired on an IncuCyte ZOOM live cell analysis system (Essen biosciences). RT-qPCR data was acquired on ABI Quantstudio 5 PCR system (Applied Biosystems).

Data analysis

Immunofluorescent and brightfield images were acquired using Zeiss Axioscan slide scanner (Z1) and Zen Blue software (v2.6) and analysed using QuPath (version 0.3.0) for automated cell counting and necrotic area analysis.
Scratch wound data were analysed on the IncuCyte proprietary scratch wound analysis software (version 2018A).
Intravital microscopy data were processed using Imaris 9.7 (Bitplane) and analysed using Imaris 9.7 (Bitplane) and ZerocostDL4mic, see Methods).
Flow cytometry data was analysed using Flowjo 10.9.0. RT-qPCR data were processed and analysed using Design & Analysis software (2.6.0, Quantstudio). Statistical analysis was performed using GraphPad Prism software version 9.4.1.
Single-nuclei sequencing analysis was performed in R (version 3.4.1), based around the following packages: Seurat R package 4.1.1, SeuratPipe R package 1.0.0, Scrublet python module 0.2.3, SoupX R package 1.5.2, Harmony R package 0.1.0. Spatial transcriptomics analysis was performed in R (version 3.4.1) using Seurat R package 4.1.1 and SPATA2 R package 0.1.0. Gene Ontology enrichment analysis was performed using the clusterProfiler R package 4.8.3. Interactome analysis was performed using CellChat R package v1.6.1. Diffusion maps and force-

directed graphs were generated in Scanpy python module v1.9. All code is made available at https://github.com/HendersonLab/LiverRegenerationAtlas.

For manuscripts utilizing custom algorithms or software that are central to the research but not yet described in published literature, software must be made available to editors and reviewers. We strongly encourage code deposition in a community repository (e.g. GitHub). See the Nature Portfolio guidelines for submitting code & software for further information.

Data

Policy information about availability of data

All manuscripts must include a data availability statement. This statement should provide the following information, where applicable:

- Accession codes, unique identifiers, or web links for publicly available datasets
- A description of any restrictions on data availability
- For clinical datasets or third party data, please ensure that the statement adheres to our policy

Our expression data will be freely available for user-friendly interactive browsing online at www.LiverRegenerationAtlas.hendersonlab.mvm.ed.ac.uk. All raw sequencing data have been deposited in the Gene Expression Omnibus (GEO Accession GSE223561). We make available as Supplementary Tables: lists of lineage-specific genes for signature analysis, lists of marker genes from clustering results, lists of gene ontology terms from enrichment analysis, lists of interactome analysis output. Source data for mouse experiments are also provided with this paper as a Supplementary Table.

The Ensembl 93 human (GRCh38) and mouse (mm10) reference genomes used in this study, originally available at https://www.ensembl.org/info/website/archives/index.html, were built by 10X Genomics and hosted at https://www.10xgenomics.com/support/software/cell-ranger/downloads#reference-downloads.

Human research participants

Policy information about studies involving human research participants and Sex and Gender in Research.

Reporting on sex and gender

Gender and age information of patients used for single nuclei-RNA sequencing are reported in SI Table 1.

Population characteristics

Specimens were obtained from the Scottish Liver Transplant Unit at the Royal Infirmary of Edinburgh, Edinburgh, UK; United States Acute Liver Failure Study Group network; Addenbrookes Hospital, Cambridge, UK; Queen Elizabeth Hospital, Birmingham, UK and University College London, London, UK. Patient gender and age for the snRNAseq datasets are reported in SI Table 1.

Recruitment

Participants were recruited based on meeting criteria for liver transplantation for acute liver failure. Healthy non-lesional liver tissue was obtained from patients undergoing surgical liver resection for solitary colorectal metastasis at the Hepatobiliary and Pancreatic Unit, Department of Clinical Surgery, Royal Infirmary of Edinburgh. Patients with a known history of chronic liver disease, abnormal liver function tests or those who had received systemic chemotherapy within the last four months were excluded from this cohort.

Ethics oversight

University of Edinburgh, UK: Local approval for procuring human liver tissue for single-nuclei RNA sequencing, spatial transcriptomics, and histological analysis was obtained from the Scotland ‘A’ Research and Ethics Committee (16/SS/0136) and the NRS BioResource and Tissue Governance Unit (study number SR574), following review at the East of Scotland Research Ethics Service (reference15/ES/0094). Written informed consent was obtained from the subject or a legally authorised representative prior to enrolment per local regulations. Acute liver failure liver tissue was obtained intraoperatively from patients undergoing orthotopic liver transplantation at the Scottish Liver Transplant Unit, Royal Infirmary of Edinburgh. Patient demographics are summarized in SI Table 1 for patients transplanted for APAP-induced ALF and nonA-E ALF. Healthy non-lesional liver tissue was obtained intraoperatively from patients undergoing surgical liver resection for solitary colorectal metastasis at the Hepatobiliary and Pancreatic Unit, Department of Clinical Surgery, Royal Infirmary of Edinburgh. Patients with a known history of chronic liver disease, abnormal liver function tests or those who had received systemic chemotherapy within the last four months were excluded from this cohort. For histological assessment of human ALF and chronic liver disease tissue, anonymized unstained formalin-fixed paraffin-embedded liver tissue sections were provided by the Lothian NRS Human Annotated Bioresource under authority from the East of Scotland Research Ethics Service REC 1, reference15/ES/0094.

United States Acute Liver Failure Study Group (ALFSG) network: This consortium of U.S. liver centers was established in 1998 to better define causes and outcomes of acute liver injury and ALF. The study protocol was approved by the local institutional review boards of the participating sites: University of Texas Southwestern Medical Center; Baylor University Medical Center, Dallas, TX; Medical University of South Carolina, Charleston, SC; University of Washington, Seattle, WA; Washington University, St. Louis, MO; University of California, San Francisco, and California Pacific Medical Center, San Francisco, CA; University of Nebraska, Omaha, NE; Mount Sinai Medical Center and Columbia University Medical Center, New York, NY; Mayo Clinic, Rochester, MN; University of Pittsburgh, Pittsburgh, PA; Northwestern University, Chicago, IL; Oregon Health Sciences Center, Portland, OR; University of California, Los Angeles, CA; University of Michigan, Ann Arbor, MI; Yale University, New Haven, CT; University of Alabama, Birmingham, AL; Massachusetts General Hospital, Boston, MA; Duke University, Durham, NC; Mayo Clinic, Scottsdale, AZ; Albert Einstein Medical Center and University of Pennsylvania, Philadelphia, PA; Virginia Commonwealth University, Richmond,VA; University of California, Davis, CA; Mayo Clinic, Jacksonville, FL; University of California, San Diego, CA; The Ohio State University, Columbus, OH; University of Kansas Medical Center, Kansas City, KS; Emory University, Atlanta, GA; University of Alberta, Edmonton, Canada. Written informed consent was obtained from the subject or a legally authorized representative prior to enrolment per local regulations. Sites obtained portions of fresh explanted liver tissue cut into 1cm3 pieces, placed into individual cryovials and stored at −80°C until requested for study. The ALFSG was supported by the National Institute of Diabetes and Digestive and Kidney Diseases (NIDDK; grant no.: U-01-58369). The samples used in this study were supplied by the NIDDK Central Repositories. This article

does not necessarily reflect the opinions or views of the NIDDK Central Repositories or the NIDDK.

University of Cambridge, UK: Patients were recruited at Addenbrooke's Hospital, Cambridge, UK with approval from the Health and Social Care Research Ethics Committee A, Office for Research Ethics Committees, Northern Ireland (ORECNI) (16/NI/0196 & 20/NI/0109). Written informed consent was obtained from the subject or a legally authorised representative prior to enrolment per local regulations. Liver tissue from patients with ALF was derived from explanted livers at the time of transplantation. All tissue samples were snap-frozen in liquid nitrogen and stored at −80°C in the Human Research Tissue Bank of the Cambridge University Hospitals NHS Foundation Trust.

University of Birmingham, UK: Human liver tissue obtained from the University of Birmingham, UK was obtained under approval by South Birmingham Ethics Committee, Birmingham, UK (reference 06/Q2708/11, 06/Q2702/61), and written informed consent was obtained from the subject or a legally authorised representative prior to enrolment per local regulations. Liver tissue was acquired from explanted livers from patients undergoing orthotopic liver transplantation at the Queen Elizabeth Hospital, Birmingham. All tissue samples were snap-frozen in liquid nitrogen and stored at −80°C before being processed and shipped by the Birmingham Human Biomaterials Resource Centre (reference 09/H1010/75; 18-319).

University College, London, UK: Human liver tissue obtained from University College, London, UK was obtained under local ethical approval (London-Hampstead Research Ethics Committee, reference 07/Q0501/50). Written informed consent was obtained from the subject or a legally authorised representative prior to enrolment per local regulations. Liver tissue (formalin-fixed, paraffin-embedded) was acquired via transjugular liver biopsy from patients presenting with acute, severe liver injury, and these patients spontaneously recovered without liver transplantation.

Note that full information on the approval of the study protocol must also be provided in the manuscript.

Field-specific reporting

Please select the one below that is the best fit for your research. If you are not sure, read the appropriate sections before making your selection.

☒ Life sciences ☐ Behavioural & social sciences ☐ Ecological, evolutionary & environmental sciences

For a reference copy of the document with all sections, see nature.com/documents/nr-reporting-summary-flat.pdf

Life sciences study design

All studies must disclose on these points even when the disclosure is negative.

Sample size

Sample size for every experiment performed and image acquired is presented in figure legends. Supplementary Table 1 also provides more detail on sample size in patient groups.
For imaging data, images in the manuscript are representative of a minimum of 4 samples within an experiment. In one experiment (Extended Data Fig. 5f) one of the human ALF aetiologies studied (HAV-induced) was n=1 (Extended Data Fig. 5f). Human sample size was determined by tissue availability.
For quantitative analyses no sample size calculation was performed, but the sample size / replicate number was chosen in order to provide sufficient data points for the statistical analyses.

Statistical significance of reported results was assessed by statistical tests as indicated in Methods section. Statistical significance is stated in each figure legend.

Data exclusions

Described in detail in Methods. Exclusion criteria were determined following initial assessment and QC of the data. Low gene expression (fewer than 1000 genes) or mitochondrial gene content <5% of the total UMI count, are indicators of outlier low quality cells and were excluded. At each stage of the analysis signature analysis was used to identify and exclude potential doublet clusters.

Replication

All experimental findings reported here were successfully replicated across multiple biological samples ('n' reported in each figure legend). All mouse experimental immunofluorescence and histology analyses were performed on a minimum of 3 liver samples to identify representative images.

Randomization

One group of randomly selected healthy liver samples and two groups of randomly selected acute liver failure samples were analysed in this study. All subsequent analyses were performed in randomly selected healthy or acute liver failure samples. For mouse experiments, age- and sex-matched mice were randomly assigned to treatment groups.

Blinding

Blinding to the origin of the tissue samples was not performed. The investigators performing the immunofluorescence staining, snRNA-seq, and RT-qPCR were different from the investigators harvesting tissue. The IVM studies were not blinded.

Reporting for specific materials, systems and methods

We require information from authors about some types of materials, experimental systems and methods used in many studies. Here, indicate whether each material, system or method listed is relevant to your study. If you are not sure if a list item applies to your research, read the appropriate section before selecting a response.

Materials & experimental systems

n/a	Involved in the study	
☐	☒	Antibodies
☐	☒	Eukaryotic cell lines
☒	☐	Palaeontology and archaeology
☐	☒	Animals and other organisms
☒	☐	Clinical data
☒	☐	Dual use research of concern

Methods

n/a	Involved in the study	
☒	☐	ChIP-seq
☐	☒	Flow cytometry
☒	☐	MRI-based neuroimaging

Antibodies

Antibodies used

All antibodies used in this work - application, supplier and product code - are listed in Supplementary Table 6 or provided in the methods section.

Validation

Antibodies listed in Supplementary Table 6 are widely used commercially available antibodies, and are validated by the companies with publications listed on the company websites. Below we provide links to these publication lists.

Validation statements from manufacturers websites:
-HNF4alpha (R&D Systems PP-H1415-00): https://www.rndsystems.com/products/human-hnf-4alpha-nr2a1-antibody-h1415_pp-h1415-00#product-citations
-HNF4alpha (Abcam ab41898): https://www.abcam.com/products/primary-antibodies/hnf-4-alpha-antibody-k9218-ab41898.html
-ANXA2 (Annexin A2) (Cell Signalling Technology 8235): https://www.cellsignal.com/products/primary-antibodies/annexin-a2-d11g2-rabbit-mab/8235
-Ki67 (Abcam ab15580): https://www.abcam.com/en-kr/products/primary-antibodies/ki67-antibody-ab15580
-Ki67 (Dako M724029-2): https://www.agilent.com/en/product/immunohistochemistry/antibodies-controls/primary-antibodies/ki-67-antigen-%28concentrate%29-76646
- CK19 (Abcam ab220193): https://www.abcam.com/products/primary-antibodies/cytokeratin-19-antibody-krt19800-ab220193.html
-RFP (Rockland Inc 600-401-379):
- https://www.rockland.com/categories/primary-antibodies/rfp-antibody-pre-adsorbed-600-401-379/
-CK18 (Abcam ab181597): https://www.abcam.com/products/primary-antibodies/cytokeratin-18-antibody-epr17347-ab181597.html
-HAL (Atlas Antibodies HPA038548):https://www.atlasantibodies.com/products/primary-antibodies/triple-a-polyclonals/anti-hal-antibody-hpa038548/
-CYP3a4 (Abcam ab3572): https://www.abcam.com/products/primary-antibodies/cytochrome-p450-3a4cyp3a4-antibody-ab3572.html
- Glutamine Synthetase (Abcam ab73593): https://www.abcam.com/products/primary-antibodies/glutamine-synthetase-antibody-ab73593.html
- BrdU (Thermo Fisher B35141): https://www.thermofisher.com/antibody/product/BrdU-Antibody-clone-MoBU-1-Monoclonal/B35141
- CD45 (Dako M0701): https://www.agilent.com/en/product/immunohistochemistry/antibodies-controls/primary-antibodies/cd45-leucocyte-common-antigen-%28concentrate%29-76507
- CD45 R&D Systems AF114): https://www.rndsystems.com/products/mouse-cd45-antibody_af114
- CD31 (Abcam ab182981): https://www.abcam.com/products/primary-antibodies/cd31-antibody-epr17259-ab182981.html
- PDGFRbeta (Abcam ab32570) https://www.abcam.com/products/primary-antibodies/pdgfr-alpha--pdgfr-beta-antibody-y92-c-terminal-ab32570.html)
- ZO-1 (Abcam ab251568): https://www.abcam.com/products/primary-antibodies/zo1-tight-junction-protein-antibody-epr19945-296-bsa-and-azide-free-ab251568.html
- F-actin (Bioss/Thermo Fisher bs-1571R): https://www.thermofisher.com/antibody/product/F-Actin-Antibody-Polyclonal/BS-1571R
- TROMA-III (DSHB ab2133570): https://dshb.biology.uiowa.edu/TROMA-III
- F4/80 BV785 (Biolegend 123141):https://www.biolegend.com/en-us/search-results/brilliant-violet-785-anti-mouse-f4-80-antibody-9919?GroupID=BLG5319&gclid=CjwKCAiAi6uvBhADEiwAWiyRdl_M3LH7PDKDh0FMChQ38p9JXsNzH8WfUR5bSbfwBkzUhgrvs7KXGhoCa04QAvD_BwE

Eukaryotic cell lines

Policy information about cell lines and Sex and Gender in Research

Cell line source(s)

Huh7 cells were procured from Cell Lines Service. Huh7 is an immortalised hepatoma cell line from a 57 year old Japanese male with well differentiated hepatocellular carcinoma.

Authentication

Huh7 cells were authenticated by the commercial supplier using STR profiling.

Mycoplasma contamination

Huh7 cells tested negative for mycoplasma contamination.

Commonly misidentified lines
(See ICLAC register)

The cell line used, Huh7, is not registered by ICLAC as commonly misidentified.

Animals and other research organisms

Policy information about studies involving animals; ARRIVE guidelines recommended for reporting animal research, and Sex and Gender in Research

Laboratory animals	Male and female mice aged 8 to 12 weeks were used for the relevant experiments. Mice were housed in conventional barrier unit facilities with conventional bedding, 12:12-hour light:dark cycle, ambient temperature control (21°C; humidity 40-60%) and access to food and water ad libitum, under pathogen–free conditions at the University of Edinburgh. Male mice were used for all experiments unless described otherwise in the figure legends. All experimental protocols were approved by the University of Edinburgh Animal Welfare and Ethics Board in accordance with UK Home Office regulations. C57BL/6JCrl mice were obtained from Charles River Laboratories (UK). mTmG (Jax 007676; B6.129(Cg)-Gt(ROSA)26Sortm4(ACTB-tdTomato,-EGFP)Luo/J) and TdTomato (Jax 007914; B6.Cg-Gt(ROSA)26Sortm14(CAG-tdTomato)Hze/J) reporter mice were obtained from Jackson Laboratories.
Wild animals	Study did not involve wild animals.
Reporting on sex	Male and female mice were used in this study.
Field-collected samples	The study did not involve samples collected from the field.
Ethics oversight	All experiments were performed in accordance with UK Home Office regulations.

Note that full information on the approval of the study protocol must also be provided in the manuscript.

Flow Cytometry

Plots

Confirm that:

☒ The axis labels state the marker and fluorochrome used (e.g. CD4-FITC).

☒ The axis scales are clearly visible. Include numbers along axes only for bottom left plot of group (a 'group' is an analysis of identical markers).

☒ All plots are contour plots with outliers or pseudocolor plots.

☒ A numerical value for number of cells or percentage (with statistics) is provided.

Methodology

Sample preparation	Primary mouse hepatocytes were isolated using in vivo perfusion digestion. Primary mouse bone marrow-derived macrophages (BMDMs) were isolated from mice femurs and differentiated for 7 days in culture with the addition of 10^4U/ml CSF1 (colony stimulating factor-1).
Instrument	BD LSR Fortessa cytometer, Becton Dickinson
Software	BD FACS Diva software was used for flow cytometry on BD LSR Fortessa equipment. Data was analysed using Flowjo v10.9.0.
Cell population abundance	n/a
Gating strategy	Differentiated primary mouse BMDMs were confirmed using flow cytometry and classed as F4/80+. F4/80+ cells that had phagocytosed dead hepatocytes were identified as Cypher-5E+. Gates and boundaries were defined by comparison to FMO and unstained samples. Representative plots of the gating strategy are available in Supplementary Table 6.

☒ Tick this box to confirm that a figure exemplifying the gating strategy is provided in the Supplementary Information.
